# Assessment of Diffuse Myocardial Fibrosis and Myocardial Oedema in Sepsis Survivors Using Cardiovascular Magnetic Resonance: Correlation with Left Ventricular Systolic Function

**DOI:** 10.3390/biomedicines13092119

**Published:** 2025-08-30

**Authors:** Ella Jacobs, Samuel Malomo, Thomas Oswald, Anthony Yip, Thomas Alway, Stanislav Hadjivassilev, Steven Coombs, Susan Ellery, Joon Lee, Claire Phillips, Barbara Philips, David Hildick-Smith, Victoria Parish, Alexander Liu

**Affiliations:** 1 Sussex Cardiac Centre, Royal Sussex County Hospital, Brighton BN2 5BE, UK; ella.jacobs@nhs.net (E.J.); s.malomo@nhs.net (S.M.); thomas.oswald@nhs.net (T.O.); t.alway@nhs.net (T.A.); stanislav.hadjivassilev@nhs.net (S.H.); steven.coombs@nhs.net (S.C.); susan.ellery1@nhs.net (S.E.); david.hildick-smith@nhs.net (D.H.-S.); victoria.parish@nhs.net (V.P.); 2 Department of Radiology, Royal Sussex County Hospital, Brighton BN2 5BE, UK; joon.lee@nhs.net; 3 Intensive Care Unit, Royal Sussex County Hospital, Brighton BN2 5BE, UK; claire.phillips20@nhs.net (C.P.); barbara.philips@nhs.net (B.P.); 4 Brighton and Sussex Medical School, Brighton BN1 9PX, UK

**Keywords:** diffuse fibrosis, myocardial oedema, heart failure, sepsis, LV dysfunction

## Abstract

**Background/Objectives**: Survivors of sepsis can develop left ventricular (LV) systolic function with focal myocardial fibrosis. The relationship between diffuse myocardial fibrosis or oedema and LV systolic function remains unknown in this patient cohort. This study sought to address this knowledge gap using cardiovascular magnetic resonance (CMR) parametric mapping methods. **Methods**: Sepsis survivors who underwent CMR at a UK cardiac centre were included. CMR images analysed include cines, native T1-mapping, native T2-mapping, and post-contrast T1-mapping. Synthetic extracellular volume (ECV) fraction was also estimated. Native myocardial T1 values, native myocardial T2 values, and ECV values were compared against LV ejection fraction (LVEF). **Results**: Of the 37 sepsis survivors (age 53 ± 16 years old; 57% males), the mean left ventricular ejection fraction (LVEF) was 55% (IQR 43–62), and 43% of the patients had LV systolic dysfunction (LVEF < 50%). Mean native myocardial T1 values were 1055 ± 65 ms (septal) and 1051 ± 60 ms (global). Mean synthetic ECV values were 0.30 ± 0.04. Mean native myocardial T2 values were 52 ± 7 ms (septal) and 53 ± 6 ms (global). Septal and global native myocardial T1 values were not significantly correlated with LVEF (rho = 0.080, *p* = 0.637; rho = 0.036, *p* = 0.831, respectively). Synthetic ECV was not significantly correlated to LVEF (rho = −0.082; *p* = 0.723). Septal and global native myocardial T2 values were weakly correlated with LVEF (rho = 0.261, *p* = 0.281; rho = 0.216, *p* = 0.375, respectively). On ROC analysis, the performance of native myocardial T1 values, ECV, and native myocardial T2 values for predicting LV dysfunction was modest (AUC: 0.53 ± 0.10, 0.54 ± 11, and 0.68 ± 0.14; all *p* > 0.05, respectively). **Conclusions**: CMR markers of diffuse myocardial fibrosis (native T1-mapping and ECV) and myocardial oedema (native T2-mapping) have weak relationships with left ventricular systolic function in this study cohort of sepsis survivors. Further work is needed to better assess the role of diffuse myocardial fibrosis and oedema in the pathophysiology of post-sepsis cardiomyopathy.

## 1. Introduction

Sepsis remains a leading global cause of morbidity and mortality, contributing to over 11 million deaths and 49 million cases each year [1,2]. A significant proportion of patients with acute sepsis can develop cardiac dysfunction [3], which is believed to be associated with the acute pro-inflammatory response and certain vascular mal-adaptations, including peripheral vasodilation, increased vascular permeability, and alterations in the cardiac pre- and after-load conditions [4,5,6]. Myocardial oedema and stress-related cardiomyopathy have been observed in patients during acute sepsis, which may be reversible and contribute to the development of acute septic cardiac dysfunction [7,8]. The cardiac dysfunction which occurs during sepsis is thought to be a reversible phenomenon [9,10]. However, more recent evidence suggests that cardiac dysfunction can be present in patients who have recovered from sepsis [11,12]. Moreover, sepsis survivors have at least a two-fold increased risk of developing long-term major adverse cardiovascular outcomes, which cannot be completely explained by the acute septic event itself nor the pre-sepsis co-morbidities [13,14]. With this prognostic threat in sepsis survivors, characterising post-sepsis cardiovascular disease and identifying possible therapeutic targets are clinical priorities.

Cardiovascular magnetic resonance (CMR) imaging provides a multi-parametric cardiac assessment of cardiac volumes and systolic function and further enables detailed characterisation of the myocardial tissue [15]. CMR is considered the reference standard modality for evaluating cardiac structure and function [15]. Late gadolinium enhancement (LGE) imaging enables the delineation of both ischaemic and non-ischaemic patterns of focal myocardial fibrosis [16,17], which has become an important part of the clinical diagnostic workflow [17,18]. Parametric mapping methods, such as native T1 (spin-lattice relaxation time) and T2 (spin-spin relaxation time) mapping, as well as extracellular volume (ECV) fractions, have complemented clinical diagnostics with the assessment of myocardial oedema and diffuse fibrosis [19,20,21].

Previous CMR data in sepsis survivors have shown the presence of left ventricular (LV) dilatation, systolic dysfunction, and a predominantly non-ischaemic distribution of myocardial fibrosis [11,22]. These findings could not be explained by the presence of significant coronary artery disease [22]. In addition to focal fibrosis, diffuse fibrosis, as detected using native myocardial T1, is also known to be higher in sepsis survivors compared to controls [11]. The relationship between diffuse fibrosis and LV function in sepsis survivors is currently unknown. Moreover, a recent study did not find significant elevations in native myocardial T2 values in sepsis survivors compared to controls [11]. Although myocardial oedema was not found to be a striking feature in the ensuing weeks after sepsis recovery, the precise contribution of oedema to LV dysfunction remains unclear [11].

This study sought to use parametric mapping methods, including T1-mapping, T2-mapping, and ECV, to assess diffuse myocardial fibrosis and oedema, with correlation of LV systolic function in sepsis survivors.

## 2. Materials and Methods

### 2.1. Study Subjects

We screened for consecutive sepsis survivors (18 years or older) who underwent clinical cardiovascular magnetic resonance (CMR) at the Royal Sussex County Hospital (Brighton, UK) between March 2015 and July 2025. Patients underwent CMR secondary to suspicions of cardiac dysfunction, e.g., on echocardiography, and/or the presence of cardiac-sounding symptoms after acute sepsis. A total of 48 sepsis survivors were found on initial screening. Patients were excluded if CMR was not tolerated (n = 1) or if CMR images did not contain T1 maps required for the study (n = 10). The final analysis included 37 sepsis survivors.

**Figure 1:**
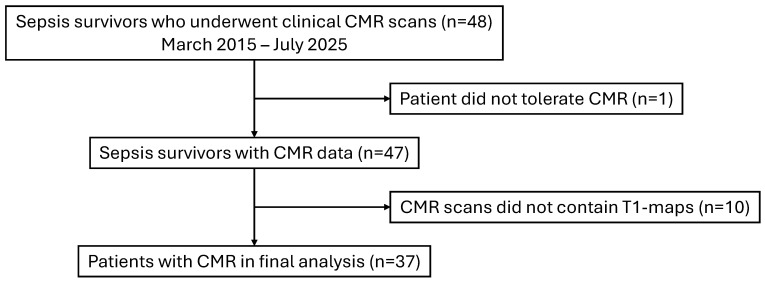
Study flowchart. CMR: cardiovascular magnetic resonance.

Figure 1 demonstrates the flowchart of patient selection and exclusion in the study.

### 2.2. Ethical Approval Statement

This retrospective study was reviewed and approved by the Research and Innovation Department of the University Hospitals Sussex NHS Foundation Trust, and informed patient consent was waived.

### 2.3. Data Collection

The clinical data of the patients were collected from the hospital’s electronic patient records. The data collected includes patient demographic data, cardiac symptoms, past medical histories, and regular medications. Samples of the data were independently validated by a second observer against the original electronic patient records for accuracy before being used for analysis.

### 2.4. Cardiovascular Magnetic Resonance (CMR)

Patients underwent CMR at 1.5 Tesla using a standard protocol containing cines, native T1-mapping, and LGE imaging [15,20]. Native T2-mapping and post-contrast T1-mapping were also performed, where able, as part of the clinical workflow [20]. Twelve patients underwent CMR with a Siemens scanner (Aera, Siemens Healthineers, Erlangen, Germany). Twenty-five patients underwent CMR with a Philips scanner (Ingenia Ambition, Philips Healthcare, Best, The Netherlands) after the opening of a new MRI centre in our hospital in 2023.

Cine images were acquired in both long- and short-axis views, using a steady-state free precession sequence [15]. Native T1-maps were acquired in a mid-ventricular short-axis slice using a vendor-provided Modified Look-Locker Inversion recovery (MOLLI) sequence with a 5s(3s)3s scheme. Native T2-mapping was performed using vendor-provided sequences (T2Map True FISP for Siemens; GraSE 9 echo sequence for Philips) in matching slice positions to the native T1-maps. LGE images were acquired in long- and short-axis views, approximately 7–8 min after an intravenous bolus of gadolinium-based contrast agent (0.1 mmol/kg; Dotarem, Guerbet, France), followed by a saline flush. Post-contrast T1-maps were acquired in matching slice positions to the native T1-maps approximately 15 min after contrast injection.

### 2.5. CMR Image Analysis

Cardiac volumes and function were analysed using commercially available software (Cvi42, Circle Cardiovascular Imaging, Calgary, AB, Canada) by clinical CMR consultants. LGE images were assessed visually by the same CMR consultants. Native T1-maps, native T2-maps and post-contrast T1-maps were analysed by an experienced observer using the commercially available Sectra Uni-view platform, as previously described [11]. Septal myocardial T1 and T2 values were assessed by manually placing a region of interest (ROI) in the septum, avoiding areas with artefacts and partial volume effects of the blood–myocardium interface [11]. Global native myocardial T1 and T2 values were also analysed by placing an ROI in a near-closed “C” shape to include as much of the global LV myocardium as possible, avoiding artefacts and the blood–myocardium interface, as previously described [11]. Samples of T1- and T2-map ROI and data were independently verified by another observer for accuracy.

Native myocardial T1 and T2 values were previously shown to be similar in control subjects scanned on both Philips and Siemens scanners in our institution [11]. Therefore, all native myocardial T1 and T2 values from both scanners in this study were pooled for analysis. Synthetic extracellular volume fraction (ECV) was assessed in patients who underwent CMR in the Philips scanner using a method previously described [19]. An ROI was in the left ventricular (LV) blood pool on native T1-maps to estimate the native T1 values of blood (T1-blood) [19]. Synthetic haematocrit was derived for MOLLI at 1.5 Tesla using the formula (922.6 × [1 ÷ T1-blood]) − 0.1668, as previously described [19].

### 2.6. Statistical Analysis

Data were checked for normality using the Shapiro–Wilk test. Parametric data were expressed as mean ± standard deviation (SD). Non-parametric data were expressed as median [interquartile range; IQR]. Parametric continuous data were compared using the independent sample *t*-test. Non-parametric data were compared using the Mann–Whitney test. Correlations between two continuous variables are assessed using Spearman’s rank correlation coefficient. The diagnostic performance of CMR parameters for predicting left ventricular dysfunction was assessed using Receiver Operating Characteristics (ROC) curves, with area under the curve (AUC) expressed with standard error of the mean (SEM); *p*-values < 0.05 are considered statistically significant. Data were analysed using commercially available software (MedCalc, version 20.104, Mariakerke, Belgium) and validated by a second observer.

## 3. Results

### 3.1. Patient Demographics Data

Of the 37 sepsis survivors (age 53 ± 16 years old; 57% males), dyspnoea was the commonest symptom (41%), followed by chest pain (24%) and palpitations (14%; Table 1). Atrial fibrillation (24%) and hypertension (24%) were the commonest co-morbidities, followed by smoking (16%), COPD/asthma (16%), and diabetes (14%; Table 1). The minority of patients had hypercholesterolaemia (8%) and chronic kidney disease (8%; Table 1).

Pneumonia was the commonest cause of sepsis (68%), and 43% of patients required management in the intensive care unit (ICU). The clinical characteristics of patients are shown in Table 1.

### 3.2. CMR Data of Study Patients

The study patients underwent clinical CMR a median of 72 days (IQR 25–123) after acute sepsis (Table 2). The LV ejection fraction (LVEF) was 55% (IQR 43–62), and the right ventricular ejection fraction (RVEF) was 52% (IQR 48–59; Table 2). LV systolic dysfunction (LVEF < 50%) was found in 42% of patients, and severe LV dysfunction (LVEF < 35%) was seen in 3% of patients (Table 2).

Evidence of LGE was found in the LV of 51% of patients, and no LGE was found in the RV in any study patient (Table 2). The septal and global myocardial native myocardial T1 and T2 values are shown in Table 2. The mean synthetic ECV was 0.30 ± 0.04 (Table 2).

### 3.3. Relationship Between Myocardial T1 and T2 Parameters with LV Systolic Function

**Figure 2:**
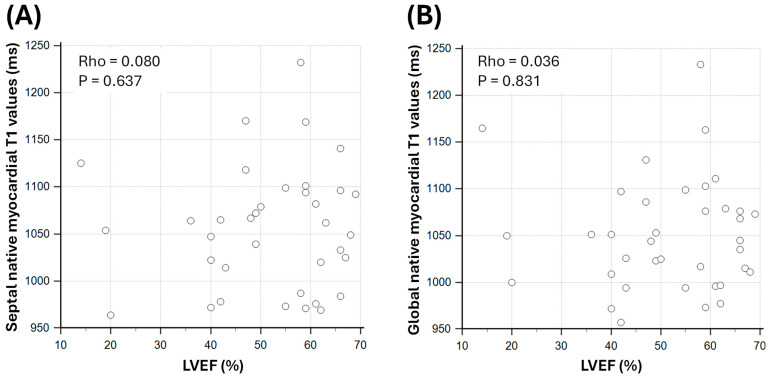
Correlation between native myocardial T1 values and LV systolic function in sepsis survivors. (**A**) shows the relationship between septal native myocardial T1 values and LVEF. (**B**) shows the relationship between global native myocardial T1 values and LVEF. LVEF: left ventricular ejection fraction; Rho: Spearman’s rank correlation coefficient.

There were weak and non-significant correlations between native septal myocardial T1 values and LVEF (Spearman’s Rho = 0.080; *p* = 0.637) and between native global myocardial T1 and LVEF (Rho = 0.036; *p* = 0.831; Figure 2).

**Figure 3:**
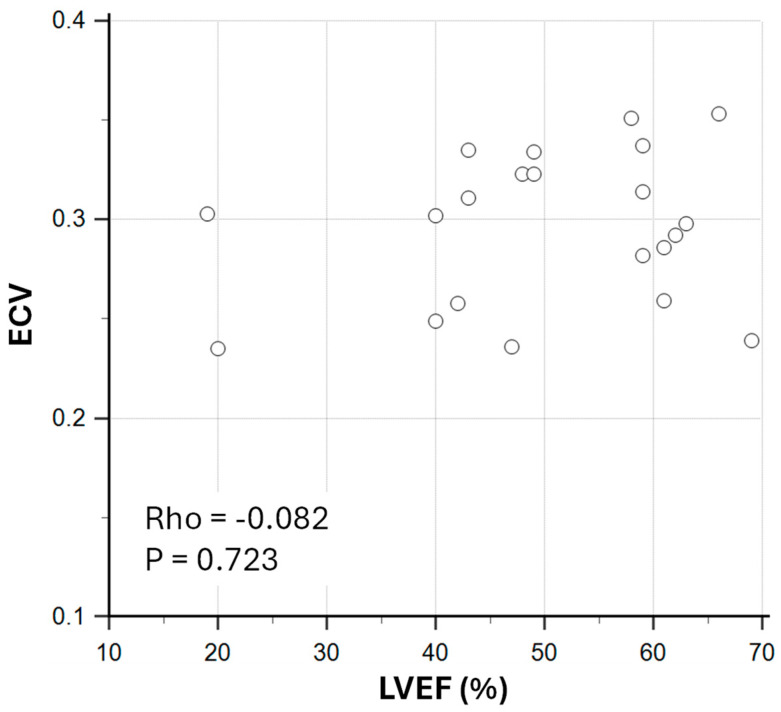
Correlation between extracellular volume fraction (ECV) and left ventricular ejection fraction (LVEF) in sepsis survivors. Rho: Spearman’s rank correlation coefficient.

There was a weak and non-significant correlation between ECV and LVEF in sepsis survivors (Rho = −0.082; *p* = 0.723; Figure 3).

**Figure 4:**
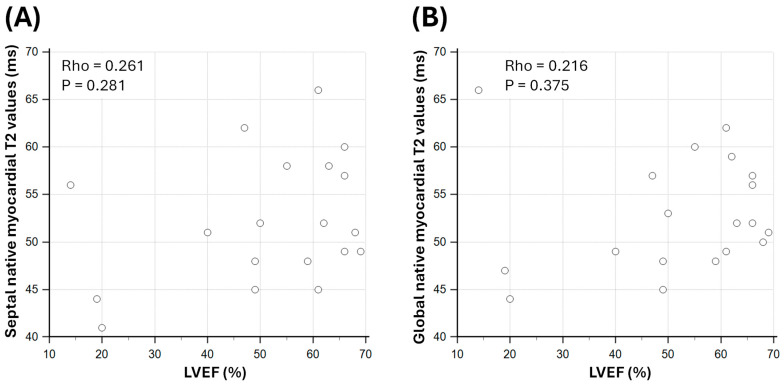
Correlation between native myocardial T2 values and left ventricular ejection fraction (LVEF) in sepsis survivors. Panel (**A**) shows the relationship between septal native myocardial T2 values and LVEF. Panel (**B**) shows the relationship between global native myocardial T2 values and LVEF. Rho: Spearman’s rank correlation coefficient.

Septal native myocardial T2 values were weakly correlated with LVEF in sepsis survivors (Rho = 0.261; *p* = 0.281; Figure 4). Global native myocardial T2 values were also weakly correlated with LVEF (Rho = 0.216; *p* = 0.375; Figure 4).

### 3.4. Predictive Value of Parametric Mapping Methods for LV Dysfunction in Sepsis Survivors

**Figure 5:**
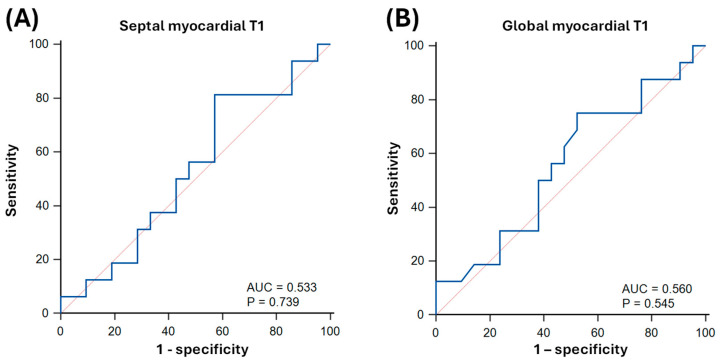
Receiver operator characteristics (ROC) curves of native T1-mapping for predicting left ventricular systolic dysfunction (LVEF < 50%) in sepsis survivors. Panel (**A**) indicates septal native myocardial T1, and panel (**B**) indicates global native myocardial T1. AUC: area under the curve.

On ROC analysis, septal native myocardial T1 achieved an AUC of 0.53 ± 0.10 (*p* = 0.739) for predicting LV systolic dysfunction as defined by LVEF < 50% (T1-cutoff: 1079 ms; sensitivity: 81% [95% CI: 54–96]; specificity: 43% [22–66]; Figure 5). Global native myocardial T1 achieved an AUC of 0.56 ± 0.10 (*p* = 0.545) for predicting LV systolic dysfunction (T1-cutoff: 1053 ms; sensitivity: 75% [48–93]; specificity: 48% [26–70]; Figure 5).

**Figure 6:**
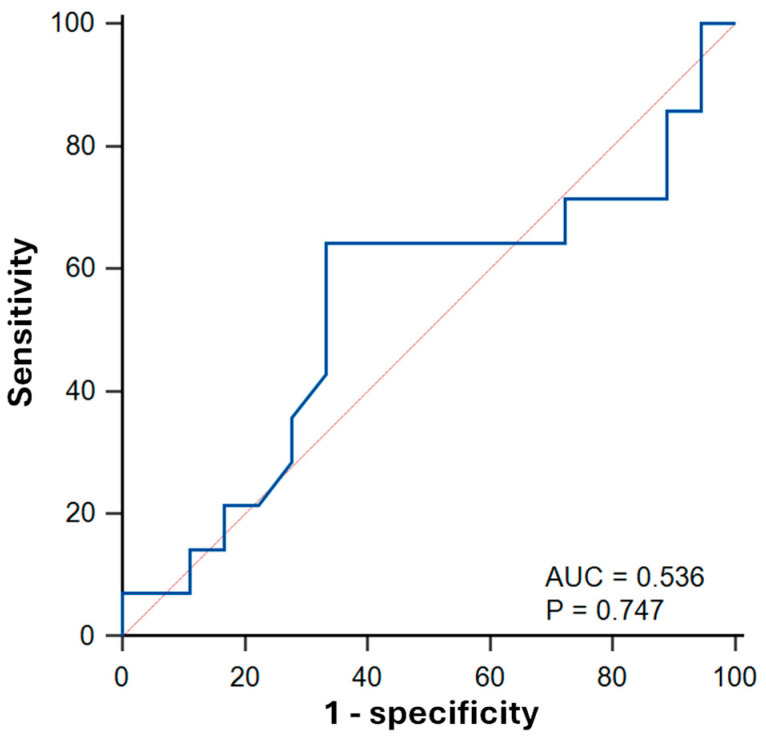
Receiver operator characteristics (ROC) curves of synthetic extracellular volume (ECV) fraction for predicting left ventricular systolic dysfunction (LVEF < 50%) in sepsis survivors. AUC: area under the curve.

Synthetic ECV achieved an AUC of 0.54 ± 0.11 (*p* = 0.747) for predicting LV systolic dysfunction (ECV cutoff: 0.30; sensitivity: 64% [95% CI: 35–87]; specificity: 67% [41–87]; Figure 6).

**Figure 7:**
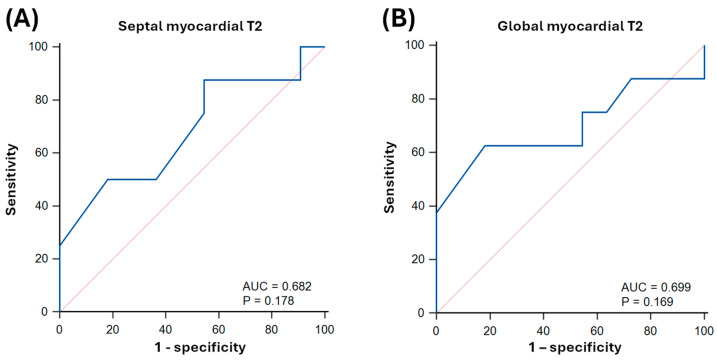
Receiver operator characteristics (ROC) curves of native T2-mapping for predicting left ventricular systolic dysfunction (LVEF < 50%) in sepsis survivors. Panel (**A**) indicates septal native myocardial T2, and panel (**B**) indicates global native myocardial T2. AUC: area under the curve.

Septal native myocardial T2 achieved an AUC of 0.68 ± 0.14 (*p* = 0.178) for predicting LV systolic dysfunction (T2 cutoff: 56 ms; sensitivity: 88% [95% CI: 47–100]; specificity: 45% [17–77]; Figure 7). Global native myocardial T2 achieved an AUC of 0.70 ± 0.14 (*p* = 0.169) for predicting LV systolic dysfunction (T2 cutoff: 49 ms; sensitivity: 63% [25–92]; specificity: 82% [48–98]; Figure 7).

**Figure 8:**
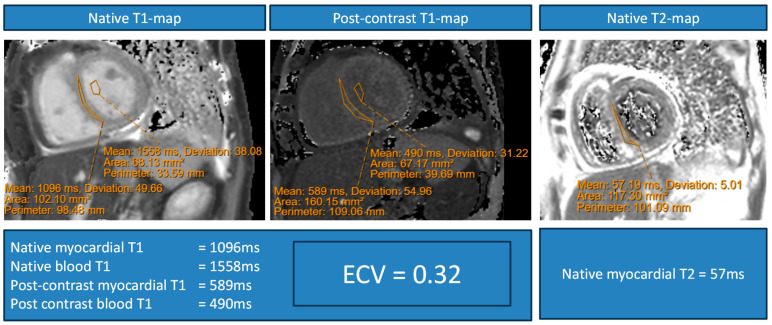
Illustrative example of a sepsis survivor with elevated native myocardial T1 values, synthetic extracellular volume (ECV) fraction, and native myocardial T2 values.

Figure 8 shows an illustrative example of a sepsis survivor with elevated native myocardial T1 values, ECV and native myocardial T2 values.

## 4. Discussion

This study characterised the association between parameters of diffuse myocardial fibrosis and myocardial oedema on CMR with LV systolic function in sepsis survivors. The main findings are as follows: (i) diffuse myocardial fibrosis, as assessed by native myocardial T1-mapping and ECV, is poorly associated with LVEF in post-sepsis patients; (ii) myocardial oedema, as assessed by native myocardial T2-mapping, was also poorly associated with LVEF in the same patients; and (iii) native myocardial T1 values, synthetic ECV, and native myocardial T2 values had overall poor performance for detecting reduced LVEF in sepsis survivors. In this study cohort, the findings generate doubt as to the ability of diffuse myocardial fibrosis and myocardial oedema alone to predict the development of cardiac dysfunction in sepsis survivors. The findings should be tested on a larger study to further assess the clinical utility of parametric mapping methods in sepsis survivors.

### 4.1. Diffuse Myocardial Fibrosis in Sepsis Survivors

Native myocardial T1 values are known to be associated with LV systolic dysfunction in patients post myocardial infarction [23] and in patients with inflammatory cardiomyopathies [24]. In patients with dilated cardiomyopathy, elevated native myocardial T1 values and ECV may indicate early myocardial fibrosis in the absence of focal scars or LGE [25]. Further, the relationship between native myocardial T1 and LV reverse remodelling after cardiac valvular surgery indicates the ability of T1-mapping methods to detect reversible pathologies in the heart [26]. To date, no study has investigated the effect of diffuse myocardial fibrosis, detected using T1-mapping methods, on left ventricular systolic function in sepsis survivors. The results of this first such study suggest that this potential link between diffuse myocardial fibrosis and cardiac systolic function is weak and non-statistically significant.

Of note, the mean native myocardial T1 values measured in the septum and globally were similar in the study cohort. This suggests that there was little regional variation in the T1 values assessed. Similarly, the correlations between septal and global native myocardial T1 values with LVEF were similarly weak. One possible explanation may be that native T1-mapping methods assess the combination of a range of myocardial properties, including healthy myocardium, diffuse interstitial fibrosis, focal fibrosis, and vascular blood content [27]. This all-encompassing ability of native T1-mapping to assess a variety of myocardial tissues also means that it may be a non-specific biomarker, which may undermine its direct correlation with left ventricular systolic function. An alternative explanation may be that a relatively small sample size in this study may have hindered the demonstration of a significant correlation between these parametric mapping methods and LVEF in this patient population. Further work using larger sample sizes is needed to evaluate the relationship between native T1-mapping and LV systolic function, including markers of more subtle LV dysfunction, such as myocardial strain analysis [28].

Whilst native T1-mapping may be prone to possible non-specific changes in the myocardial tissue of sepsis survivors, ECV may be more specific for the assessment of extracellular pathologies [29,30]. The use of synthetic ECV, with a surrogate for haematocrit derived from blood pool T1, is well documented [19,29,31]. However, the lack of significant correlation between synthetic ECV and LVEF suggests that diffuse myocardial fibrosis may not directly dictate the development of cardiac dysfunction in sepsis survivors. Previous autopsy studies have shown evidence of diffuse interstitial fibrosis in patients who succumbed to sepsis [32]. However, there is a lack of cardiac function data in these patients during life [32]. ECV values can also be influenced by the presence of focal fibrosis and extracellular blood volume [27], meaning that it may also be a non-specific marker of fibrosis in the heart. Further work is needed to better characterise the effect of ECV on cardiac function in larger studies of sepsis survivors.

### 4.2. Myocardial Oedema in Sepsis Survivors

Previous data using CMR to study post-sepsis patients suggested that myocardial oedema may not be a prominent feature some time after the acute sepsis has subsided [11]. Indeed, native myocardial T2 values in sepsis survivors are generally higher than those in non-septic controls; however, this difference was not found to be significant [11]. One explanation may be that myocardial inflammation and oedema have settled down after the acute episode. However, without serial CMR studies in this patient cohort, it remains hypothetical whether native T2-mapping can detect changes in myocardial inflation.

In this study, native myocardial T2 values were not significantly associated with LV systolic dysfunction. This suggests that myocardial oedema may have a lesser role to play in the development of cardiac dysfunction after acute sepsis. Shorter native myocardial T2 values are known to be correlated with a greater chance of LV dysfunction remodelling in dilated cardiomyopathy after treatment with guideline-directed medical therapy [33]. T2 values may also be sensitive to microvascular changes in relation to inflammation [34]. Whether the native T1 values in this study detected multiple pathologies in the myocardium of sepsis survivors remains unclear. Although considered a biomarker less dependent on the presence of myocardial fibrosis, the predictive value of native myocardial T2 for LV dysfunction requires further investigation in larger studies.

### 4.3. Limitations and Future Directions

This is a retrospective UK study in a single cardiac centre, which would be vulnerable to sampling bias, and the applicability of the findings needs to be tested in different patient populations and multi-centre studies. The fact that most patients had cardiac-sounding symptoms or suspicions for heart failure as indications for undergoing CMR scans means that there is also likely a degree of selection bias by virtue of an increased pre-test probability of the patient having a cardiac pathology. A future study in sepsis survivors on an all-comer basis may further inform on the clinical applicability of parametric mapping for detecting cardiac dysfunction. The study used LVEF as the sole outcome of LV dysfunction, which may not be sensitive to more subtle changes in cardiac function [35]. Future studies using LV strain as a marker of cardiac dysfunction may provide further insights into its relationships with diffuse myocardial fibrosis and oedema. The CMR scan was performed at a variety of times after the acute sepsis episode in patients. Future studies with CMR performed at uniform timepoints post-sepsis and with serial follow-up imaging may provide further information on the changes in myocardial T1 and T2 values in relation to cardiac dysfunction. The effect of guidelines-directed medical therapy for heart failure on markers of myocardial fibrosis and oedema could not be tested in this study owing to the lack of serial CMR scans. Future studies are needed to assess this effect and build on existing evidence [12,36]. The effect of cardiac biomarker levels and acute septic cardiac dysfunction on imaging markers of fibrosis and oedema in sepsis survivors should be studied in future research. Furthermore, the effect of vasopressor and inotropic support administered to patients during acute sepsis may influence the degree of cardiac dysfunction and fibrosis after recovery. This question should also be answered in future studies.

## 5. Conclusions

CMR markers of diffuse myocardial fibrosis (native T1-mapping and ECV) and myocardial oedema (native T2-mapping) have weak relationships with left ventricular systolic function in this study cohort of 37 sepsis survivors. Further work and larger studies are needed to better assess the role of diffuse myocardial fibrosis and oedema in the pathophysiology of post-sepsis cardiomyopathy.

## Figures and Tables

**Table 1:** Clinical characteristics of patients.

	Patients (n = 37)
Age, years	53 ± 16
Male	20 (57)
BMI, kg/m^2^	25 ± 6
Causes of sepsis	
Pneumonia	25 (68)
Gastroenterological	4 (11)
Unknown origin	3 (8)
Soft tissue/bacteraemia/abscess	5 (14)
ICU care requirement	16 (43)
Intubation	5 (14)
Vasopressor support	6 (16)
Inotropic support	5 (14)
Symptoms	
Dyspnoea	15 (41)
Chest pain	9 (24)
Palpitations	5 (14)
Co-morbidities	
Atrial fibrillation	9 (24)
Hypertension	9 (24)
Smoking (Ex-/current)	6 (16)
COPD/Asthma	6 (16)
Diabetes	5 (14)
Hypercholesterolaemia	3 (8)
Chronic kidney disease	3 (8)
Pre-sepsis history of IHD	2 (5)
Stroke/TIA	1 (3)
Pre-sepsis history of heart failure	0 (0)
Medications	
ACEi/ARB/ARNI	20 (54)
Beta-blockers	19 (51)
MRA	19 (51)
SGLT2-Inhibitor	16 (43)
Statins	12 (32)
Anticoagulation	10 (27)
Loop diuretics	8 (22)
Anti-platelet agents	7 (19)

ACE: angiotensin-converting enzyme; ARB: angiotensin receptor blocker; ARNI: angiotensin receptor-neprilysin inhibitor; BMI: body mass index; COPD: chronic obstructive airways disease; ICU: intensive care unit; IHD: ischaemic heart disease; MRA: mineralocorticoid receptor antagonist; and SGLT-2: sodium–glucose co-transporter-2. Continuous variables are displayed as mean ± SD. Categorical variables were displayed as numbers (%).

**Table 2:** Cardiovascular magnetic resonance (CMR) data of study patients.

	Patients (n = 37)
Day from sepsis event to CMR	72 [25–123]
CMR volumes and function	
LV EDVi, mL/m^2^	91 [78–107]
LV ESVi, mL/m^2^	39 [28–57]
LV SVi, mL/m^2^	48 ± 12
LV EF, %	55 [43–62]
LV EF <50%	16 (42)
LV EF <35%	3 (8)
RV EDVi, mL/m^2^	86 ± 23
RV ESVi, mL/m^2^	40 [32–46]
RV SVi, mL/m^2^	45 ± 13
RV EF, %	52 [48–59]
LV mass index, g/m^2^	64 [57–73]
LGE data	
LV LGE present	19 (51)
Subepicardial/Mid-wall	16 (43)
Subendocardial	3 (8)
RV LGE present	0 (0)
Native myocardial T1 values	
Septal	1055 ± 65
Global	1051 ± 60
Native myocardial T2 values	
Septal	52 ± 7 (n = 19)
Global	53 ± 6 (n = 19)
Synthetic ECV	0.30 ± 0.04 (n = 21)

ECV: extracellular volume fraction; EDVi: end-diastolic volume index; EF: ejection fraction; ESVi: end-systolic volume index; LGE: late gadolinium enhancement; LV: left ventricular; RV: right ventricular; SVi: stroke volume index. Continuous variables are displayed as mean ± SD or median [interquartile range]. Categorical variables were displayed as numbers (%).

## Data Availability

The patient data in this study cannot be shared in the public domain; reasonable requests for the data should be sent to the corresponding author.
